# Amoxicillin and Clarithromycin Use and Delirious Mania in an Adolescent With Recurrent Catatonia and Psychosis

**DOI:** 10.7759/cureus.112367

**Published:** 2026-07-09

**Authors:** Laura Pulido, Matthew D Jandrisevits, Sophia M Valenti, Colleen K Manak, Scott J Sandage

**Affiliations:** 1 Child and Adolescent Psychiatry, Medical College of Wisconsin, Milwaukee, USA; 2 Psychiatry and Behavioral Medicine, Medical College of Wisconsin, Milwaukee, USA; 3 Medicine, Medical College of Wisconsin, Milwaukee, USA; 4 Psychiatry, Medical College of Wisconsin, Milwaukee, USA; 5 Psychiatry and Behavioral Medicine/Child and Adolescent Psychiatry, Medical College of Wisconsin, Milwaukee, USA

**Keywords:** amoxicillin therapy, antibiotics-induced psychiatric symptoms, cannabis-induced psychosis, catatonia with psychosis, clarithromycin, delirious mania, h. pylori treatment, recurrent psychosis, triple therapy for h. pylori

## Abstract

The intersection of pediatric psychiatry and infectious disease can present complex diagnostic challenges, particularly when symptoms of organic medical etiology mimic severe psychiatric disorders. This case study analyzes the clinical trajectory of an adolescent patient who developed delirious mania - a subtype of catatonia combining manic, delirious, and psychotic features - following the administration of clarithromycin and amoxicillin for *Helicobacter pylori (H. pylori)* gastritis. Initially presenting with psychosis and delirium attributed to a prior Coronavirus Disease 2019 (COVID-19) infection and recurrent cannabis use, the patient underwent six hospital admissions over four months. While the patient’s initial symptoms were thought to be likely due to cannabis-induced psychosis, the emergence of treatment-resistant delirious mania during her sixth admission led to treatment shifts that resulted in symptom improvement. This case review proposes a methodological framework for mitigating “anchoring bias” and identifying iatrogenic or other triggers in treatment-refractory psychiatric presentations. Ultimately, this work emphasizes the critical need for continuous medical re-evaluation in psychiatric settings as well as demonstrates that even common antibiotic regimens, like “triple therapy” for *H. pylori*, can precipitate severe neuropsychiatric syndromes in vulnerable adolescents.

## Introduction

The differential diagnosis of acute psychiatric symptoms in adolescents often requires navigating complex presentations that include biological predispositions, substance use histories, and potential underlying medical conditions. Recent literature has increasingly highlighted how systemic physiological stressors, such as Coronavirus Disease 2019 (COVID-19), can produce profound neuropsychiatric complications, including delirium with severe agitation in pediatric patients. For example, a 2022 two-person case series was among the first to identify that COVID-19 could produce delirium with agitation in adolescent patients [1]. Notably, an adolescent in that case series who also used cannabis was noted to have a longer hospital course, more medications at higher dosages, and more use of five-point restraints than a patient who had never used cannabis.

When these novel viral exposures are combined with known risk factors, such as a family history of schizophrenia and daily cannabis use, clinicians frequently arrive at diagnoses like substance-induced psychosis or first-episode primary psychosis. However, the reliance on these established narratives can lead to diagnostic overshadowing in which anomalous symptoms are erroneously attributed to the pre-existing psychiatric diagnosis. This is known as anchoring bias, in which physicians focus on a single piece of information to formulate a diagnosis without sufficiently adjusting to additional information [2].

The current case study examines the apparent development of anchoring bias in the treatment of a complex presentation that included psychosis, delirium, mania, and catatonia in the context of substance use and COVID-19 infection.

A description of this case begins with a clear symptom taxonomy, rooted in the Diagnostic and Statistical Manual, fifth edition, text revision (DSM-V TR) [3]. Psychosis is defined as an altered perception of reality, featuring delusions, auditory hallucinations, and disorganized behavior, arising from both psychiatric and organic causes [3]. Delirium, or encephalopathy, is a syndrome of brain dysfunction characterized by altered attention and cognition that is acute in onset and often fluctuating throughout the day [3]. Mania involves elevated mood, euphoria, irritability, grandiosity, and decreased need for sleep, typically associated with mood disorders but occasionally induced by substances or medications [3]. Catatonia represents a syndrome of motor dysfunction encompassing rigidity, posturing, and agitation, as well as other odd signs that may be elicited on an exam [3,4]; this is often screened for and tracked for severity with the Bush-Francis Catatonia Rating Scale [5]. Finally, while not in the DSM-V TR, “delirious mania” is a rare, high-acuity subtype of catatonia that synthesizes catatonic, manic (± psychotic), and delirious symptoms with rapid onset [6].

Management of multi-morbid psychiatric presentations can be challenging for psychiatrists on a pediatric consultation liaison service; this is even more challenging when cases involve comorbid substance use, organic factors, and adverse reactions to medical treatments. Standard psychiatric diagnostic processes are highly susceptible to anchoring bias, wherein clinicians fixate on a patient’s initial presentation, such as cannabis-induced psychosis, and fail to adequately adjust their differential diagnosis when new symptoms, such as acute mania, emerge. Existing guidelines lack pediatric-specific frameworks for rapidly identifying rare, medication-induced psychiatric syndromes, particularly those triggered by common non-psychotropic medications such as antibiotics. When an adolescent patient suddenly exhibits treatment-refractory severe agitation, grandiose delusions, and autonomic instability, clinical decisions can default to escalation of psychopharmacological interventions rather than pause to investigate newly introduced medical treatments. This oversight can result in negative clinical outcomes as well as potentially the prolonged use of violent restraints and intensive care unit admissions, as observed in the case under review.

There are limited case reviews that address the issue of anchoring bias in the treatment of adolescent patients that present with psychosis, delirium, mania, catatonia, substance use, and antibiotic therapy. This case study of an adolescent female is presented to propose a cautious approach for evaluating atypical psychiatric presentations. Specifically, the purpose of this case study is to provide a comprehensive analysis demonstrating that the combination of amoxicillin and clarithromycin, commonly prescribed for *Helicobacter pylori* (*H. pylori*) gastritis, can precipitate delirious mania in an adolescent, highlighting a critical iatrogenic risk. Additionally, this case study offers guidance for medical and psychiatric teams to prevent anchoring bias and enforce the re-evaluation of medical and pharmacological etiologies whenever a patient’s established psychiatric symptoms do not respond to initial treatment.

## Case presentation

Written informed consent was obtained from the patient (now an adult) for publication. Gina (name and age altered to protect privacy) was a previously healthy 17-year-old honors student who demonstrated generally typical premorbid functioning. She had a family history of schizophrenia in a second-degree family member and reportedly used cannabis daily. Over the course of this case review, she was hospitalized six times in the span of four months. She was admitted four times to a pediatric hospital, where the psychiatry consultation-liaison (C-L) psychiatry team was consulted on her each time. There were also two psychiatric hospitalizations during that span. While all hospitalizations are referenced in this case study, this article primarily focuses on treatment provided at the pediatric hospital that involved the psychiatry C-L service.

**Figure 1:**
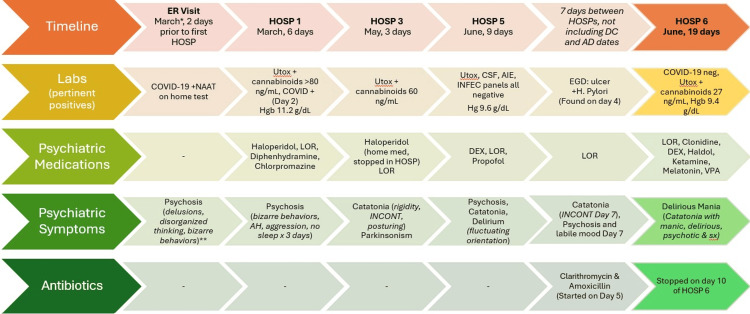
Timeline of major events. Labs, psychiatric medications, psychiatric symptoms, and antibiotics are shown respective to the timeline. *The months of the events have been changed for patient privacy, but the chronological relationships of the events to each other are accurate. **Italicized text in parentheses () in the "Psychiatric Symptoms" section is an example of the symptoms Gina had of the psychiatric diagnosis immediately before the parenthetical text. AD: admission; AIE: autoimmune encephalitis; AH: auditory hallucinations; COVID-19: coronavirus disease 2019; CSF: cerebrospinal fluid; DC: discharge; DEX: dexmedetomidine; ER: emergency room; HOSP: hospitalization; INCONT: urinary incontinence; INFEC: infectious (viral, bacterial, and parasitic); LOR: lorazepam; NAAT: nucleic acid amplification test; sx: symptoms; Utox: urine toxicology; VPA: valproic acid; x: for.

Figure 1 provides a visual timeline for the treatment course. Gina first presented to the pediatric emergency department (ED) with altered mental status in the context of cannabis use as well as COVID-19 exposures in her household the prior week. She was medically evaluated and discharged home with a recommendation to initiate outpatient mental health services. However, Gina’s altered mental status worsened, including reported auditory hallucinations, bizarre and aggressive behaviors, and three consecutive days without sleep. Two days later, she presented again to the pediatric ED. Gina was then admitted for a first episode of psychosis and delirium, and a hospital-obtained COVID-19 Nucleic Acid Amplification Test (NAAT) was positive on admission. The initial differential diagnosis included substance-induced versus COVID-19-related psychosis and delirium. Her urine toxicology panel was significant for cannabinoids >80 ng/mL. Her hemoglobin was noted to be in a mildly anemic range at 11.2 g/dL. The medical team initially diagnosed Gina with unspecified psychosis. Subsequently, the medical team consulted with the psychiatry C-L service. The psychiatry C-L service recommended haloperidol, lorazepam, diphenhydramine, and chlorpromazine throughout that six-day hospitalization.

Once medically stable, Gina was transferred from the pediatric hospital to an outside psychiatric hospital to complete acute psychosis stabilization. This was identified as her second admission in her overall course, and she was discharged from that facility after approximately seven days with a prescription for haloperidol and lorazepam and outpatient psychiatric follow-up.

Within the next two months, Gina again presented at the pediatric hospital for her third overall admission. Gina’s symptoms included new catatonia (diagnosed using DSM 5-TR criteria [3] with some assistance from the Bush Francis Catatonia Rating Scale, BFCRS, for screening and severity tracking [5]) in the context of rapid weaning of lorazepam, as well as parkinsonism associated with her haloperidol treatment for her psychosis. Gina’s urine toxicology screen again was positive for cannabis but was down trending, now below 60 ng/mL. The psychiatry C-L team managed Gina’s haloperidol and adjusted her lorazepam, and her symptoms improved. She was discharged after three days with a recommendation to follow up with her outpatient psychiatrist.

Gina’s fourth admission occurred after she presented at a psychiatric hospital for psychosis, delirium, and catatonia with significant agitation. Staff at the psychiatric hospital reportedly observed blood in the toilet of Gina’s bathroom. Additionally, Gina’s worsening and severe agitation resulted in daily staff injuries and equipment damage at the psychiatric hospital, to the point where she was placed in a unit wing with no other patients for safety reasons. She required transfer to the pediatric hospital for sedation as well as a medical work-up.

The transfer from the psychiatric hospital to the pediatric hospital was Gina’s fifth overall admission. Gina was admitted to the Pediatric Intensive Care Unit (PICU) for a dexmedetomidine infusion to provide sedation that protected against severe agitation and for a comprehensive medical work-up for bleeding. Interestingly, at that time, Gina’s urine was negative for traces of cannabis. She received a broad work-up for psychosis, including cerebrospinal fluid analysis for infectious encephalitis; autoimmune encephalitis panels; expanded substance panels; sexually transmitted diseases; infectious serum panels (viral, bacterial, and parasitic); and imaging of her brain; and chest, abdomen, and pelvis. All were negative for obvious organic causes of psychosis. After several days of her treatment regimen, including continued dexmedetomidine infusion and lorazepam, Gina’s symptoms stabilized. For her bleeding and anemia (hemoglobin 9 g/dL), she had an esophagogastroduodenoscopy (EGD) done the day prior to discharge, during which biopsy samples were taken to be analyzed over the next few days. Since the EGD only showed a few small erosions in her stomach, she was discharged home on a proton-pump inhibitor (PPI) for possible gastritis and lorazepam for mild catatonia.

Four days after discharge, the EGD biopsy results confirmed *H. pylori* gastritis as the cause of her bleeding and subsequent anemia. Five days after discharge, she was started on amoxicillin 1000 mg twice daily and clarithromycin 500 mg twice daily for *H. pylori* gastritis, in addition to the daily PPI started in the prior hospitalization, to complete her “triple therapy” for *H. pylori*. Three days after starting those antibiotics, Gina was brought to the emergency room after her mom noted newly slowed movements, irrational behavior, and urinary incontinence. This resulted in her sixth admission, which occurred at the pediatric hospital.

While in the pediatric ED for her sixth admission, Gina was noted to be severely agitated, highly irritable, and speaking rapidly about paranoid delusions. When the C-L psychiatry team (LP, CKM, and SJS) examined her, they also noted auditory hallucinations, disorganized thinking, and significant psychomotor agitation, which indicated psychotic symptoms. They noted some grandiose delusions and labile mood, potentially indicative of elevated mood. They noted automatic obedience and echolalia, which indicated worsening catatonia, and finally noted that her orientation to the date and the situation fluctuated, indicating delirium/encephalopathy. Gina was diagnosed with unspecified psychosis, catatonia, and delirium and was admitted to a general medical inpatient unit. A basic medical work-up was done on admission, including a urine toxicology that was only positive for cannabinoids (27 ng/mL), a COVID-19 NAAT that was negative, and a hemoglobin level of 9.4 g/dL, which was only slightly lower than the prior admission. Her EKG, urine pregnancy test, comprehensive metabolic panel, salicylate level, acetaminophen level, and alcohol ethyl were all normal, negative, or unremarkable.

By day four of her sixth hospitalization, Gina's agitation had escalated despite treatment with lorazepam for catatonia and clonidine for delirium and agitation management. With 30 minutes of sleep the night prior, ongoing grandiose delusions, and new sexually inappropriate behaviors, she was diagnosed with delirious mania - due to clear and worsened manic symptoms in addition to her catatonic, psychotic, and delirious symptoms - and transferred to the PICU.

While in the PICU, she was given a dexmedetomidine drip for several days, as this had helped resolve her severe agitation during her fifth admission - when it was initially prescribed due to some team members’ (SJS, CKM) prior experience with treating severe COVID-19-related psychotic agitation [1].

After several days of little to no response to the dexmedetomidine, other measures were tried: exchanging guanfacine for clonidine while continuing her dexmedetomidine drip for delirium treatment and general sedation, adding intravenous propofol for sedation, and even once trialing ketamine for sedation. All of these interventions failed to reduce the severity of her agitation, which threatened her and others’ safety. Verbal and physical de-escalation techniques by appropriately trained staff were used multiple times a day, and even so, physical restraint devices were used near daily due to her elopement attempts, sudden rages damaging equipment in her room, and generally severe agitation.

On day 10, after no significant improvement and ongoing safety issues, one of the C-L psychiatrists (SJS) re-initiated a clinical assessment that included a fresh, thorough chart review for any recent changes in Gina’s medical history compared with prior hospitalizations due to the unusual development of manic symptoms, rapid decompensation compared with prior hospitalizations, and poor response to previously helpful treatments. Gina’s prescription for antibiotics a few days prior to her sixth admission was the only notable change, but it was the key information that led to a shift in the team’s conceptualization of her current symptoms as well as her treatment plan. A literature review at that time subsequently found several reports of clarithromycin, with or without amoxicillin, leading to “manic psychosis” in adults [7]. There were no identified similar case reports in adolescents that were found at the time, but given the shared features of “manic psychosis” in adult case reports and Gina’s current presentation, the C-L psychiatry team advised the primary critical care team to discontinue both amoxicillin and clarithromycin immediately. Gina’s psychiatric symptoms trended toward improvement soon after this shift in her care plan. By day 12, Gina was started on valproic acid every six hours. Over days 13-19, she continued to improve and was soon weaned off of dexmedetomidine. She was able to be discharged home on lorazepam (for catatonia) and valproate (for mood stabilization) on day 19 with minimal signs of catatonia, as well as resolution of her manic, delirious, and psychotic symptoms. Gina subsequently engaged in regular outpatient psychiatry appointments with a diagnosis of Schizophrenia Spectrum and Other Psychotic Disorder, which is the most updated iteration of "unspecified psychosis" per the Diagnostic and Statistical Manual, version 5, text-revision (DSM-V TR) [3].

## Discussion

Gina was hospitalized six times over the course of this case study with severe and escalating psychotic symptoms, as well as catatonia in the last three admissions. Interestingly, while Gina’s initial psychosis was highly suspected to be cannabis-induced, her episodes did not always correlate with an increase in urine cannabinoids. In her fifth admission to our pediatric hospital, Gina’s urine screen was actually negative for cannabinoids, and yet she still struggled with agitation, psychosis, and catatonia. When the psychiatry C-L team pivoted away from assuming etiology based on substance use or premorbid psychiatric illness, Gina’s treatment shifted, and she experienced rapid symptom improvement during her sixth (index) admission. Despite this difficult course that spanned months, Gina recovered to the extent that her symptoms were managed at the outpatient level without further hospitalization for over one year.

Psychosis frequently occurs as a symptom associated with a wide range of psychiatric, neurologic, and medical conditions, making it often difficult to elucidate the cause of psychosis in the clinical setting. The same can be said for catatonia and for delirium as well. In Gina’s case, her initial and sudden onset of psychotic symptoms without a prodrome of negative symptoms led the treating team to initially deduce either a COVID-19 or substance-induced process, or both. These were reasonable conclusions. A six-month retrospective cohort study based on the electronic health records of 236,379 patients diagnosed with COVID-19 found a 33.62% incidence of neurological or psychiatric diagnoses, including a 1.4% incidence of psychosis, in 2021 (2.77% in an ICU) [8]. In that study, when compared with matched controls with other respiratory viruses, the hazard ratio was 1.32. In Gina’s case, the team understood that her COVID-19 infection could either predispose her to psychosis or precipitate the first break in addition to the recent cannabis exposure.

Cannabis use poses an even higher risk of psychosis than COVID-19. A European study of 901 patients with first-episode psychosis found that daily cannabis use was associated with increased odds of psychotic disorder compared with never-users (OR: 3.2; 95% CI: 2.2-4.1) and especially with high-potency cannabis (OR: 4.8; 95% CI: 2.5-6.3) [9]. Gina had been a chronic user of cannabis, so whatever cannabis exposure she had immediately prior to her first hospitalization may have been the precipitant to her first break on top of predisposing factors (family history and COVID-19 exposure). Interestingly, though her urine toxicology was negative during the fifth admission, it was positive at 27 ng/mL during her sixth admission. Though the level was quite low compared to her other results across all admissions, the psychoactive cannabinoid tetrahydrocannabinol (THC) is metabolized by the CYP 3A4 enzyme, and her newly started clarithromycin, a strong CYP 3A4 inhibitor, may have raised THC levels in Gina’s serum, thus leading to worsening side effects of cannabis [10]. In Gina’s case, a broad work-up for psychosis completed during her fifth admission helped rule out other organic causes of psychosis, catatonia, and delirium, such as autoimmune encephalitis, systemic autoimmune disease, infectious, oncologic, and metabolic causes. Since this low level of cannabis, even with CYP 3A4 inhibition, would not have been expected to cause agitation at the level of severity she exhibited, the team shifted toward seeking an alternative clinical hypothesis, which led to a breakthrough in Gina’s treatment.

In this case, our “anchoring bias” was rooted in the presumed cannabis-induced psychosis noted in multiple prior hospitalizations, rather than the chief complaint on the index admission, which is how the term was described in a large cross-sectional study published in 2023 [2]. All the same, it was only in moving beyond this “anchoring bias” that the team was able to consider how broader medications, including antibiotics, can induce psychiatric syndromes. In particular, clarithromycin - part of the “Triple Therapy” for *H. pylori* gastritis - has been found to have GABA-A antagonistic activity that has been hypothesized to relate directly to epileptogenicity and neurotoxic effects [11]. Additionally, the CYP 3A4 inhibition of clarithromycin may increase cortisol and prostaglandin levels, which can lead to neuropsychiatric side effects [10,11]. Ultimately, Gina’s history of COVID-19 infection and cannabis use likely contributed to her prior presentations with catatonia, agitation, and psychosis but proved to be “red herrings” in her final admission. When the C-L psychiatry team broadened the assessment beyond the known psychiatric history, the team identified that the patient’s *H. pylori* antibiotics had been started a few days prior to hospitalization. Reports of psychosis and other psychiatric sequelae in adult patients prompted the removal of these antibiotics, which ultimately led to the resolution of her delirious mania symptoms. The clear resolution of delirious mania following the discontinuation of antibiotics was notable, as she discharged home nearly at baseline from the index hospitalization without ongoing antipsychotic use.

One challenge encountered in this case was that an initial literature search did not yield any case reports that described *H. pylori* antibiotic-induced psychosis in adolescents at the time of treatment. Additionally, adult literature did not include terms like “manic psychosis” or “transient psychosis” that were specifically relevant to this case. A meticulous and time-consuming literature review was required to identify a presentation and treatment that matched this case of delirious mania. This sort of detailed reading often takes more time than we can afford at the point of care in clinical scenarios such as ours with dangerous and difficult-to-control agitation happening at all hours of the day. Secondly, since the body of literature pertaining to delirious mania and/or “triple therapy” neuropsychiatric side effects in adolescents was so small, the authorship team found it helpful to review case reports with broader (mainly adult) patient populations. In this way, the current case report adds to the body of literature by describing delirious mania, specifically from clarithromycin ± amoxicillin (part of *H. pylori* “triple therapy”), in a pediatric patient. The hope is that this case may help future medical teams struggling with a similarly ill pediatric patient.

An obvious limitation to our case report is the fact that the patient’s urine was still positive for cannabis during the index admission. One could argue that we had not completely ruled out cannabis-induced psychosis during this admission, and perhaps it was the cannabis exposure, more so than the antibiotics, that led to delirious mania. Another limitation is that, since Gina already had a psychiatric history and biological predispositions for psychiatric symptoms, this case is less generalizable to other cases with antibiotic “Triple therapy" without a psychiatric history. Delirious mania, especially as an extreme form of psychiatric adverse effects, is very rare. Finally, since multiple treatments were trialed, including starting valproic acid around the same time as her antibiotics were discontinued, it is difficult to clearly show that her improvement was from antibiotics being removed and not from valproic acid, though the level of serum valproic acid before discharge was 77 ug/mL, which is subtherapeutic for acute mania.

Finally, we do acknowledge a weakness in the report in terms of not stating Gina’s Bush-Francis Catatonia Rating Scale [5] scores, which would be a useful way to look at the severity and phenotype of catatonia at various time points. This was purposely left out, as the BFCRS was not consistently used to track symptoms across hospitalizations and to not detract from the main objectives of this particular case report.

## Conclusions

In an adolescent patient presenting with refractory delirious mania following multiple hospital admissions for psychosis with or without catatonia, symptoms improved after discontinuation of clarithromycin and amoxicillin, which had been prescribed for comorbid *H. pylori* infection as part of the common “triple therapy” treatment for this infection. This temporal association between the addition of the antibiotics and the onset of delirious mania was quite strong. So our primary aim with this case report was to add to the literature documentation of delirious mania in an adolescent in correlation with amoxicillin and clarithromycin. Our secondary aim for this report is to demonstrate why it is important that clinicians maintain clinical curiosity about underlying medical causes of “psychiatric” syndromes, such as delirium or catatonia, particularly when symptoms do not improve with psychiatric treatments as expected or have additional elements that do not follow the established pattern. It is understandable that treatment teams may fall prey to the “anchoring bias” of prior diagnoses (as we did) and cease assessing for medical causes of psychiatric presentations that feel familiar. However, whenever there is a new or atypical presentation of psychiatric symptoms, it behooves us to closely and systematically review the patient’s recent medical history for changes in medications, supplements, illness states, lab values, infectious status, substance use, travel history, or other contributors to find a potential correlate for the change. If no obvious changes are found with these initial steps, it may then be worthwhile to repeat prior medical work-up, even if previously unremarkable, to properly rule out medical etiologies for the change when nothing else appears to have precipitated it.
